# The Accuracy of a Web-Based Visual Acuity Self-assessment Tool Performed Independently by Eye Care Patients at Home: Method Comparison Study

**DOI:** 10.2196/41045

**Published:** 2023-01-25

**Authors:** Janneau Claessens, Juultje van Egmond, Joukje Wanten, Noël Bauer, Rudy Nuijts, Robert Wisse

**Affiliations:** 1 Department of Ophthalmology University Medical Center Utrecht Utrecht Netherlands; 2 University Eye Clinic Maastricht Maastricht University Medical Center+ Maastricht Netherlands; 3 Xpert Clinics Oogzorg Zeist Netherlands

**Keywords:** eHealth, telemonitoring, telemedicine, telehealth, visual acuity, eye care, ophthalmology

## Abstract

**Background:**

Telehealth solutions can play an important role in increasing access to eye care. Web-based eye tests can enable individuals to self-assess their visual function remotely without the assistance of an eye care professional. A web-based tool for self-assessing visual acuity (VA) has previously been studied in controlled, supervised conditions. The accuracy of this tool when performed independently by patients in their home environment, using their own devices, has not yet been examined.

**Objective:**

The objective of this paper was to examine the accuracy of a web-based tool with respect to measuring VA in ophthalmic patients in their home environment, compared with a conventional in-hospital assessment using a Snellen chart (the gold standard).

**Methods:**

From April through September 2020, consecutive adult patients with uveitis at the University Medical Center Utrecht, the Netherlands, performed the web-based VA test at home (the index test) before their upcoming conventional VA assessment at the hospital (the reference test). The agreement between the 2 tests was assessed by the Bland-Altman analysis. Additional analyses were performed to investigate associations between clinical characteristics and the accuracy of the web-based test.

**Results:**

A total of 98 eyes in 59 patients were included in the study. The difference in VA between the index and reference tests was not significant, with a mean difference of 0.02 (SD 0.12) logMAR (*P*=.09) and 95% limits of agreement of –0.21 to 0.26 logMAR. The majority of the differences (77%) fell within the predetermined acceptable deviation limit of 0.15 logMAR. In addition, no patient characteristics or clinical parameters were found to significantly affect the accuracy of the web-based test.

**Conclusions:**

This web-based test for measuring VA is a valid tool for remotely assessing VA, also when performed independently by patients at home. Implementation of validated web-based tools like this in the health care system may represent a valuable step forward in revolutionizing teleconsultations and can provide individual patients with the opportunity to self-monitor changes in VA. This is particularly relevant when the patient’s access to ophthalmic care is limited. Future developments should focus on optimizing the testing conditions at home to reduce outliers.

## Introduction

The sharp and sudden decrease in health care access during the COVID-19 pandemic underlined the importance of telehealth services for remote patient monitoring. But also in the postpandemic world, telehealth can play an important role in achieving universal health access [1,2]. Considering eye care, web-based eye tests can enable individuals to self-assess their visual function remotely using their own electronic devices, without the assistance of an eye care professional. Several research teams around the globe have been evaluating and implementing a smartphone-based eye test in community- or school-based screening for visual impairment [3-8]. But also in eye care practices, web-based eye tests are of great value, as they can enrich teleconsultations by providing eye care professionals and patients with a quantifiable measurement of visual function without a clinic visit [9].

Visual acuity (VA) is the ability of the eye to correctly distinguish details of an object at a given distance [10]. It is one of the key parameters of an ophthalmic (ie, eye care) patient’s evaluation and is conventionally assessed at a clinic using a white chart displaying black optotypes—typically letters or symbols—that patients should correctly identify from a standardized distance [11]. Multiple tools for self-assessing VA have been introduced over the last decade, though many lack clinical validation [12,13]. Before implementing a telehealth tool in clinical practice, validation research and certification is needed [14]. The medtech company Easee B.V. (Amsterdam, the Netherlands) developed the world’s first Conformité Européenne–certified web-based assessment of refractive error and VA, in collaboration with our clinical specialists (RW). The accuracy of this web-based test at assessing VA has been previously validated in controlled, *supervised* settings in healthy individuals 25 (SD 5) years of age [15], and in a relatively young cohort of keratoconus (a disease affecting the structure of the eye’s cornea) patients 26 (SD 5) years of age [9], with robust results, particularly in the higher VA range. We hypothesize that this self-assessment of VA can serve as a reliable and feasible substitute for a conventional in-hospital assessment, including in older patients and patients with limited mobility. Nevertheless, the ability of this web-based test to provide reliable estimates of VA when performed by patients in *unsupervised*, in-home settings has not yet been examined. This study evaluates the accuracy of the web-based VA test when performed independently by ophthalmic patients at home.

## Methods

### Ethical Approval

This study was approved by the local medical ethics committee (METC Utrecht, the Netherlands; review number: 21-072) and performed in accordance with Dutch privacy laws and the Declaration of Helsinki. A study invitation letter, informed consent form, and return envelope were sent by mail. The letter contained comprehensive information about the study, including a statement that there was no (financial) compensation for, or benefits related to, participation. Contact details of the research team were given to discuss any questions or concerns. Written informed consent was obtained from all participants in this study: patients were instructed to sign the informed consent form and send it using the provided return envelope when willing to participate. All study data were coded and stored in a database only accessible to the research team. Data collected by Easee B.V. were stored on General Data Protection Regulation–compliant and Health Insurance Portability and Accountability Act–compliant servers located within the European Union. This paper was written in adherence to the STARD 2015 guidelines for reporting diagnostic accuracy studies [16].

### Study Design and Patient Recruitment

This method comparison study was conducted at the University Medical Center Utrecht, the Netherlands, from April through September 2020. In this period, many nonurgent outpatient visits were either rescheduled or postponed. We therefore focused on recruiting patients with uveitis (an inflammatory eye condition), as their outpatient visits were considered essential and not likely to be canceled. In addition, a large number of these patients previously provided consent to be approached for participation in future research.

All consecutive adult patients scheduled to visit our uveitis clinic were invited. Those who were willing to participate were requested to perform the web-based test at home before their hospital visit. We instructed patients to reperform the web-based test, or reach out to the study team, whenever they experienced a change in VA before their hospital visit. For this study, we excluded patients who did not perform an in-hospital test within 14 days of completing their web-based test and patients who changed their glasses or contact lens prescription or who reported a change in their VA between the web-based and in-hospital tests without repeating the web-based eye test.

### Web-Based VA Assessment (Index Test) and Conventional VA Assessment (Reference Test)

**Figure 1:**
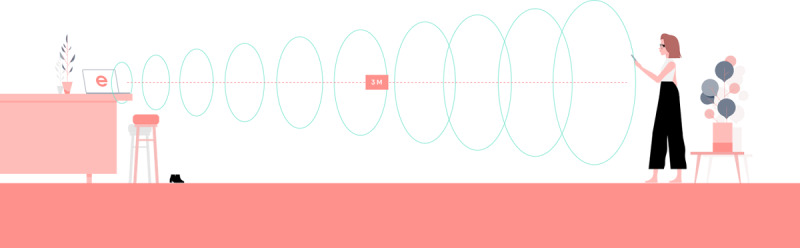
Schematic diagram depicting the web-based test (not to scale). During the test, the patient is instructed to stand 3 m away from the screen and use the smartphone to control the test.

**Figure 2:**
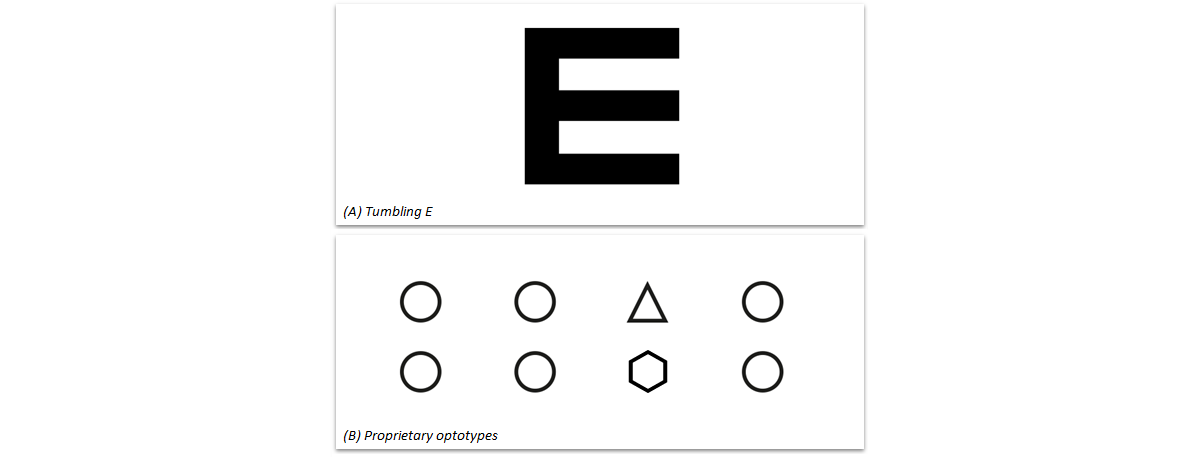
Different optotypes presented on the computer or tablet screen during the test. (A) Four kinds of rotations of this optotype will be displayed on the smartphone screen. The patient will be asked to select the one that is identical to the optotype presented on the computer or tablet screen. (B) A row of 4 numbers (1-4) will be displayed on the smartphone screen. The patient will be asked which of the 4 optotypes presented on the computer or tablet screen is different.

Patients were instructed to perform the web-based test in their home 1 to 14 days before their hospital visit. This test is accessible via a dedicated URL via the institution’s patient portal, and users must have a computer or tablet, a smartphone, and an internet connection to perform the test. In brief, the smartphone serves as a remote control through which the users submit their input a distance of 3 m from the computer or tablet screen (Figure 1). Audio instructions guide them through the test. During the test, the computer or tablet screen displays a sequence of optotypes—varying in size—that the patients must correctly identify (Figure 2). A calibration step in the setup phase of the tool reassures that the displayed optotypes are correctly sized, regardless of the screen dimensions of the patient’s own device. The VA score will be determined based on the answers provided by the user.

Before the test starts, one can manually select which eye to measure. Users will be requested to cover the contralateral eye with their hand during the assessment. All participants were instructed to complete the web-based eye test once for each eye and wear their standard spectacles or contact lenses for distance vision, if applicable, while performing the assessment. If the web-based test was performed multiple times, we collected the most recent outcomes only.

Conventional VA measurements were performed during the hospital visit by an eye care professional using a Snellen chart at 6 m (the standardized distance for this chart). During this assessment, the patients also wore their standard spectacles or contact lenses, if applicable, and the clinical staff were blinded with respect to the outcomes of the patient’s previously performed web-based test.

### Data Collection

The outcomes of the web-based test were collected by Easee B.V., the developer of the test. The following clinical data were collected from the patient’s electronic health record: sex, age, ophthalmic diagnosis, medical history, use of medication, and VA measurements; in addition, because we included patients with uveitis, we also collected their uveitis classification and symptoms associated with active uveitis. All ophthalmologists at our ophthalmology clinic use the Standardization of Uveitis Nomenclature (SUN) classification criteria [17]. Specifically, uveitis disease activity was classified based on vitreous haze (VH), anterior chamber cell count (ACC), optical coherence tomography, and fluorescent angiography and dichotomized as “inactive” (both ACC and VH ≤0.5 and not considered active by a specialist) or “active” (ACC>0.5, VH>0.5, or considered active by a specialist).

### Statistical Analysis

Our main outcome was the accuracy of the web-based test for measuring VA, compared with the conventional in-hospital VA assessment performed within 14 days. The patient’s web-based VA was reported in logarithm of the minimum angle of resolution (logMAR) units, and the in-hospital Snellen decimal score was converted to logMAR units. Measurement accuracy is expressed as the mean difference between the 2 assessments, with 95% limits of agreement (95% LoA; ie, the range within 95% of the differences between the 2 assessments is included). This methodology was first introduced by Bland and Altman and is commonly used to evaluate the agreement between 2 measurements on a continuous scale [18]. Varying outcomes are common when repeatedly performing a VA test in an individual patient [19-21]. In line with an authoritative cross-sectional study performed in a large eye clinic using various charts and observers, we considered an absolute difference between tests >0.15 logMAR to be clinically relevant [19].

The minimum VA score that can be measured using the web-based test is 0.05 Snellen decimal (1.3 logMAR); thus, scores lower than this value are reported as “<0.05 Snellen decimal (>1.3 logMAR).” Because the exact VA in these patients was unknown, patients with a VA score >1.3 logMAR were not included in the Bland-Altman analysis but were descriptively analyzed as a subgroup.

We also performed a subgroup analysis to investigate the possible association between clinical characteristics and agreement between the index and reference test outcomes. Specifically, we analyzed patients with an absolute difference >0.15 logMAR (ie, “underperformance” of the web-based test) versus patients with an absolute difference ≤0.15 logMAR (ie, “good performance” of the web-based test). Differences between these groups were analyzed using the chi-square test or independent-sample Student’s *t* test.

A multivariable generalized estimating equation (GEE) model was used to assess the association between clinical variables and the VA outcome of both tests. The GEE model was designed to correct for bilateral disease, age, sex, use of a mydriatic agent, ocular comorbidity that can affect VA, symptoms associated with uveitis activity, and the interval (in days) between the index and reference tests.

## Results

### Included Patients

A total of 59 patients met all of the inclusion criteria. Our analysis included 98 eyes (20 patients performed the web-based assessment for only 1 eye). Patient recruitment is depicted in Multimedia Appendix 1, and a participation bias toward younger patients is appreciated (mean age of patients not willing to participate vs included patients: 53, SD 19 vs 47, SD 15 years). The clinical characteristics of the study population are summarized in Tables 1 and 2. Consistent with our overall uveitis population, approximately two-thirds (68%) of the participants were female. The mean interval between the index test and the reference test was 4.8 (SD 2.7) days. At the time of their visit to our ophthalmology clinic, 27% of eyes had symptoms of potentially active uveitis, including ocular pain, floaters, photophobia, and vision loss. On the basis of the SUN classification [17], 73% of patients had nonanterior uveitis and 97% had a chronic disease course. At the time of their visit, 25% of eyes were classified as having “active inflammation” of uveitis, whereas the other 75% were classified as “inactive.”

**Table 1:** Clinical characteristics of the study population (patients: N=59).

Clinical characteristics		Values
Age (years), mean (SD)		47 (15)
**Sex, n (%)**		
	Male	19 (32)
	Female	40 (68)
Interval between tests (days), mean (SD)		4.8 (2.7)
**Ophthalmic medication^a^, n (%)**		
	Mydriatics	4 (7)
	Other	45 (76)
**Uni- or bilateral uveitis, n (%)**		
	Unilateral	16 (27)
	Bilateral	43 (73)
**Anatomical classification^b^, n (%)**		
	Anterior	16 (27)
	Nonanterior	43 (73)
**Uveitis course^b^, n (%)**		
	Acute	2 (3)
	Chronic	57 (97)

^a^Use of ophthalmic medication at the time of the in-hospital appointment.

^b^According to the Standardization of Uveitis Nomenclature classification [17].

**Table 2:** Uveitis characteristics of the study population.

Uveitis characteristics (per eye)		Eyes (N=98), n (%)
**Activity of uveitis**		
	Inactive^a^	73 (75)
	Active^b^	24 (25)
	Not reported	1 (1)
Visual acuity influencing comorbidities at the time of appointment^c^		30 (31)
Anamnestic symptoms of active uveitis^d^		26 (27)

^a^When anterior chamber cell count (ACC) and vitreous haze (VH) is ≤0.5 and not called active by the ophthalmologist.

^b^When ACC or VH is ≥1 or called active by the ophthalmologist.

^c^Including (secondary) cataract, keratitis, scleritis, corneal lesion, or history of pars plana vitrectomy.

^d^Symptoms associated with active uveitis: ocular pain, floaters, photophobia, and visual loss.

### Accuracy of the Web-Based VA Test

**Figure 3:**
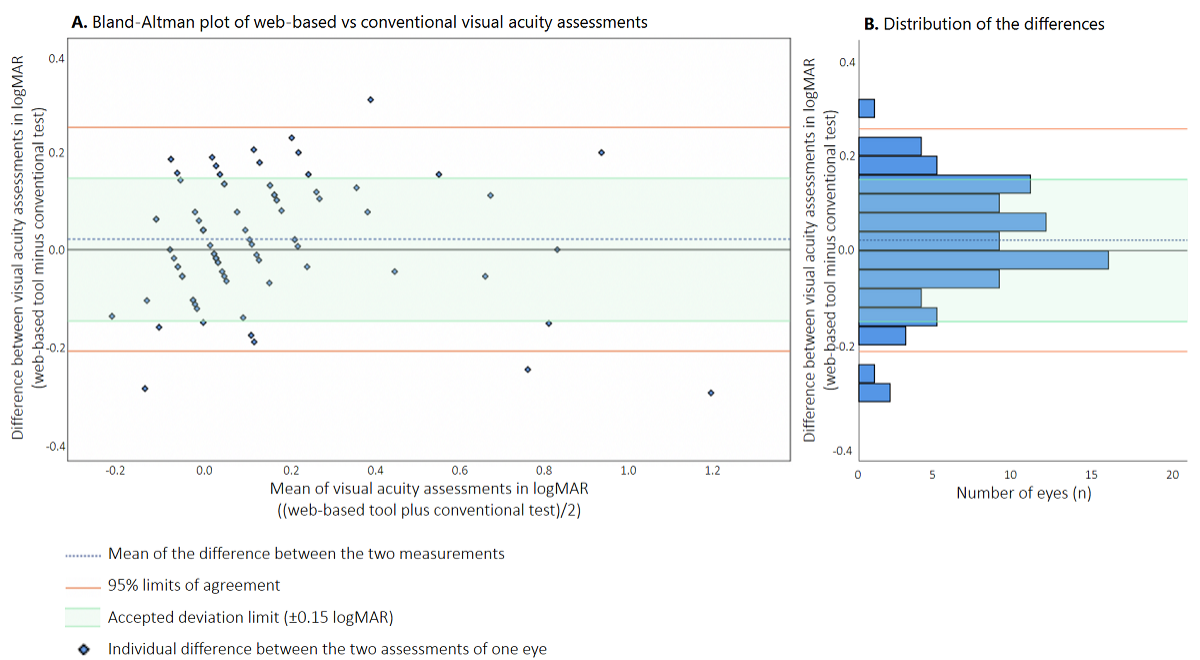
(A) Bland-Altman plot comparing the results of the web-based VA test and the results of the conventional VA test. Each symbol indicates an individual eye. (B) Distribution histogram summarizing the data shown in panel A. logMAR: logarithm of the minimum angle of resolution; VA: visual acuity.

The mean VA measured using the web-based test was 0.12 (SD 0.25) logMAR (0.86, SD 0.37 Snellen decimal), which was similar to the conventional in-clinic assessment (0.10, SD 0.25 logMAR; 0.89, SD 0.32 Snellen decimal; mean difference: 0.02, SD 0.12 logMAR, *P*=.09). The Bland-Altman plot (Figure 3A) summarizes the difference between the 2 tests for 91 eyes with a web-based VA of ≥0.05 Snellen decimal. The 95% LoA ranged from –0.21 to 0.26 logMAR, with no indication of a proportional bias. Overall, 70 of these 91 eyes (77%) fell within the predetermined acceptable deviation limit of ±0.15 logMAR. As shown in Figure 3B, a distribution histogram of the difference between VA values reveals that the peak difference was close to zero (ie, virtually no difference between test results). We found similar results when we performed a Bland-Altman analysis on the left eyes only (mean difference 0.01, SD 0.13 logMAR; 95% LoA –0.23 to 0.26 logMAR) and on the right eyes only (mean difference 0.03, SD 0.11 logMAR; 95% LoA –0.19 to 0.25). Finally, an indication of the reliability of the web-based test is found in the concordance of 2 separate measurements within the same individual: subjects who performed the web-based test for both eyes (n=39 patients) showed a similar accuracy for both separate monocular measurements.

#### Subgroup Analysis of Patients With a Poor Web-Based VA Score

Seven eyes had a VA score below the minimum detectable limit of the web-based test (ie, <0.05 Snellen decimal). For 6 of these eyes, however, VA was indeed measured correctly using the web-based test, as the corresponding VA measured using the conventional reference test was also <0.05 Snellen decimal. Remarkably, one eye with a VA score <0.05 Snellen decimal based on the index test was found to have a VA of 0.4 Snellen decimal based on the conventional test; upon inquiry, however, the patients reported that the web-based test was difficult to perform, indicating that the VA measured using the web-based test likely did not represent their actual VA.

#### Subgroup Analysis of Good Performance Versus Underperformance on the Web-Based Test

The results of our subgroup analysis comparing eyes in which the web-based test had good performance (n=70 eyes) and eyes in which the web-based test underperformed (n=21) are shown in Table 3. We found no significant difference between subgroups with respect to any of the clinical characteristics analyzed.

**Table 3:** Subgroup analysis of “good performance” vs “underperformance” of the web-based test.

Characteristics		Overall	logMAR^a^ difference ≤0.15 (“good performance”)	logMAR^a^ difference >0.15 (“underperformance”)	*P* value	
Number of eyes, n		91	70	21	N/A^b^	
Age (years), mean (SD)		45 (15)	44 (15)	48 (15)	.24	
**Sex, n (%)**						.66
	Male	31 (34)	23 (33)	8 (38)		
	Female	60 (66)	47 (67)	13 (62)		
Interval between tests (days), mean (SD)		4.6 (2.6)	4.6 (2.8)	4.7 (2.0)	.89	
Visual acuity influencing comorbidities^c^, n (%)		26 (29)	17 (24)	9 (43)	.10	
Anamnestic symptoms at the time of appointment^d^, n (%)		23 (25)	17 (24)	6 (29)	.69	
**Ophthalmic medication use, n (%)**						.53
	Mydriatic	7 (8)	6 (9)	1 (5)		
	Other	69 (76)	54 (77)	15 (71)		
	None	15 (17)	10 (14)	5 (24)		
**Uveitis anatomical classification^e^, n (%)**						.08
	Anterior	27 (30)	24 (34)	3 (14)		
	Nonanterior	64 (70)	46 (66)	18 (86)		
**Uveitis course^e^, n (%)**						.43
	Acute	2 (2)	2 (3)	0 (0)		
	Chronic	89 (98)	68 (97)	21 (100)		
**Activity of uveitis at the time of appointment, n (%)**						.19
	Inactive^f^	68 (75)	50 (71)	18 (86)		
	Active^g^	23 (25)	20 (29)	3 (14)		

^a^logMAR: logarithm of the minimum angle of resolution.

^b^N/A: not applicable.

^c^Including (secondary) cataract, keratitis, scleritis or corneal lesions at time of appointment, or history of pars plana vitrectomy.

^d^Symptoms associated with uveitis: ocular pain, floaters, sensitivity to light, and visual loss.

^e^According to the Standardization of Uveitis Nomenclature classification [17].

^f^When anterior chamber cell count (ACC) and vitreous haze (VH) ≤0.5 and not called active by the ophthalmologist.

^g^When ACC or VH is ≥1 or called active by the ophthalmologist.

#### Generalized Estimating Equation Analysis

The GEE analysis revealed no significant association between VA and any of the clinical variables examined (Multimedia Appendix 2). Specifically, we found no clinical factors—uveitis-related or otherwise—that appeared to affect VA measured using both tests or either test individually.

## Discussion

### Principal Findings

In this study, we examined the accuracy of a web-based tool for self-assessing VA when performed remotely by ophthalmic patients. Our results indicate that ophthalmic patients can use this web-based tool to estimate VA independently in their own home, which is particularly advantageous when access to the clinic is limited. We found a clinically negligible mean difference of 0.02 (SD 0.12) logMAR between the web-based test results and in-hospital chart assessments, and the majority of the comparisons (77%) fell within the conventional and predetermined acceptable deviation limit of 0.15 logMAR [19]. This negligible mean difference indicates that there is no fixed bias, meaning that the web-based test does not systematically over- or underestimate VA. The distribution of the differences (indicated by the 95% LoA) slightly exceeded the predetermined acceptable limit, pointing out that some of the patients had a larger difference between the 2 assessments than expected based on normal measurement variation. Subgroup analyses did not identify clinical characteristics that affected agreement between the 2 tests.

For this study, we focused on patients with uveitis. Importantly, however, tools for measuring VA are considered to be universally applicable, regardless of any underlying ocular conditions. It is therefore reasonable to speculate that the web-based tool’s accuracy observed in patients with uveitis will be similar when used by similar-aged (ie, similarly digitally proficient) and similarly visually proficient patients with other ocular conditions.

When comparing between different tests of VA, 2 important phenomena should be taken into account. First, a certain degree of variability is inevitable when repeatedly measuring VA in the same eye, even in the absence of any clinical changes between tests, due to the psychophysical nature of VA testing. Outlier measurements occur even in controlled in-hospital settings, owing to patients’ behavioral factors such as concentration, fatigue, and a low intrinsic motivation. Studies that focused on test-retest variability using Snellen VA charts reported that 95% LoA ranged from ±0.18 logMAR (using the single-letter method) to ±0.33 logMAR (using the line assignment method) from the mean difference [20,22]. The line assignment method (in which the test is terminated when at last half of the letters are misread) remains the most popular method in clinical practice, despite the introduction of more reliable alternatives such as the Early Treatment Diabetic Retinopathy Study (ETDRS) chart [21,23,24]. Based on the 95% LoA values (±0.24 logMAR from the mean difference), the precision of the remote web-based test used in our study appears to be fairly similar to the precision of conventional VA testing using Snellen charts. Secondly, differences in VA are inevitable when using 2 different types of VA charts [19,25,26]. In the web-based test, patients were presented with a combination of tumbling E optotypes and proprietary optotypes (Figure 2), whereas for the conventional examination, a Snellen letter chart was used. Thus, a conversion effect may have contributed—at least in part—to the observed differences in VA between the web-based test and the conventional test.

### Comparison With Prior Work

We previously examined the accuracy of the web-based VA test in healthy individuals [15] and in patients with keratoconus [9]. Compared with our previous results, the distribution of differences in VA was smaller. We attribute this to measuring corrected (better) VA in this study, whereas our previous studies measured uncorrected (poorer) VA. Measurement accuracy is known to be suboptimal in these poorer VA ranges, particularly when using a Snellen chart [21]. Interestingly, this was the first time the web-based tool was used by patients in a completely unsupervised situation, namely the patient’s own home in which lighting and test conditions were controlled exclusively by the patient. We believe that this greatly increases the generalizability of the outcomes of our study. Notably, the fact that the test was unsupervised did not appear to affect its overall accuracy.

There are many other telehealth tools for self-assessing visual function available in app stores or on the World Wide Web, though many of these have not been validated [12,13]. A well-established tool is the “Peek Acuity” smartphone app, which was first introduced by Bastawrous et al in 2015 [25]. This tool has been evaluated by various research teams [3-6,27]. In a recent validation study, conducted among hospital employees, a mean difference of 0.01 logMAR (95% LoA: –0.27 to 0.29 logMAR) was reported when compared with a conventional clinical chart. Another application that has been evaluated multiple times is the “EyeChart” app [28,29]. Tiraset et al evaluated the “EyeChart” application in ophthalmic patients and reported similar results (mean difference: 0.01 logMAR; 95% LoA: –0.21 to 0.19 logMAR) as our study [29]. Overall, our findings with respect to the web-based tool developed by Easee are similar to the mean differences and 95% LoA observed using these other VA self-assessment tools. Importantly, note that these other tools were evaluated in controlled settings. In addition, these tools require a person to hold the smartphone or tablet (presenting the optotypes) and submit answers on the touch screen, while the patient stands at a distance from this screen. The present study focuses on the self-administration of a web-based VA test at home. The Easee eye test is highly intuitive, and a paired smartphone is used as a remote control, negating the need for assistance.

### Future Perspectives

Our results indicate that an unsupervised, remote web-based VA test can serve as a validated option for measuring VA in ophthalmic patients who are both willing and able to perform the self-test, including patients with a complex ocular disease such as uveitis. Hence, the web-based test can enrich teleconsultations in ophthalmic care and create opportunities for patients with a chronic condition to self-assess their VA at home when they suspect that their visual function might be deteriorating. We learned from our clinic’s patient board that patients consider this form of self-control to be important. We do not claim that this web-based tool can fully replace a comprehensive ophthalmic examination, nor do we claim that it by itself is sufficient for adequately following *all* ophthalmic patients. Based on our subgroup analyses, it is not possible to use clinical parameters to preselect patients for whom the web-based test is considered unsuitable.

It is important to note that some of the included patients performed poorly on the web-based test. We did not identify clinical factors, such as uveitis activity, that affect measurement accuracy. Interestingly, we found that most patients performed the web-based test with equal reliability for both eyes. It is therefore reasonable to speculate that the test’s performance is affected the most by behavioral factors such as the patient’s competence using digital devices, their intrinsic motivation, and the environmental conditions when performing the test such as lighting and setup. Given these factors, we strongly recommend introducing telehealth tools for self-assessing visual function on an individual level and in close consultation with the patient. Adequate patient instructions (and compliance with these instructions) are essential to the tool’s success, especially in unsupervised settings. The web-based test flow is highly intuitive, though future changes to the tool should focus on optimizing the testing conditions at home, for example, by using the webcam to provide feedback regarding lighting conditions and the patient’s distance from the screen. Still, outlier measurements might occur, as these also occur in in-hospital settings using conventional charts. We propose that whenever web-based test outcomes are suspected to be invalid, patients should be instructed to retest under optimal testing conditions. This is not different from what we would do in an in-hospital setting using conventional charts.

### Limitations

Several considerations warrant further discussion. First, we included only patients who were willing and able to perform the web-based eye test at home. As the participation flow demonstrates (Multimedia Appendix 1), this study design resulted in a participation bias in favor of younger patients, who are potentially more comfortable using digital devices. The accuracy of the web-based test may be poorer in patients who are less competent or comfortable using digital devices, as tests may be performed incorrectly. However, it is important to note that successful completion of the web-based test does not necessarily indicate adequate performance. In our study, we included poorly performing patients in our analyses, as information regarding outliers is important for interpreting the web-based test’s accuracy and identifying patient characteristics that may be correlated with poor performance. Notwithstanding, we strongly recommend that future studies evaluate the performance of the web-based tool in older, less digitally competent patient populations. Second, all patients were first-time users of the web-based test. We recommend future studies to determine whether directions of variations within patients are similar when repeating web-based self-assessments at different time points. It is important to understand the test-retest variability of the web-based test and to identify whether learning effects can be appreciated, indicated by a better accuracy when repeatedly performing the test.

### Conclusions

In summary, we report that the web-based VA test, performed unsupervised and independently at home, provided a reliable measure of VA in the majority of patients in our study. The in-home assessment appears a feasible substitute for a conventional Snellen chart assessment at the clinic. Implementing this web-based test into the health care system enriches teleconsultations by providing patients with the tools they need for self-monitoring, which is particularly valuable when access to hospital care is limited. We found no clinical characteristics that significantly affected the accuracy of the web-based test. Outliers beyond the clinically acceptable range of –0.15 to +0.15 logMAR were identified, which we consider a common feature of VA testing and attributed to behavioral and environmental factors. Future developments of the web-based test should focus on optimizing testing conditions at home to reduce the potential effects of these factors.

## Appendix

Multimedia Appendix 1 Flowchart showing patient recruitment, including the mean (±SD) ages of the indicated groups.

Multimedia Appendix 2 Multivariable GEE analysis to investigate the associations between clinical variables and the visual acuity outcomes in logMAR.

Multimedia Appendix 3 Data set generated and analyzed during this study.

## Abbreviations

ACC: anterior chamber cell count
GEE: generalized estimating equation
logMAR: logarithm of the minimum angle of resolution
SUN: Standardization of Uveitis Nomenclature
VA: visual acuity
VH: vitreous haze 95% LoA: 95% limits of agreement

## Data Availability

The data set generated and analyzed during this study is included in Multimedia Appendix 3.
