# Supplementary material for: The Accuracy of a Web-Based Visual Acuity Self-assessment Tool Performed Independently by Eye Care Patients at Home: Method Comparison Study
Source: JMIR Form Res. 2023 Jan 25;7:e41045. doi: 10.2196/41045 (PMC9909522; doi:10.2196/41045)
Supplement: Multimedia Appendix 1 [file formative_v7i1e41045_app1.pdf]

Multimedia Appendix 1

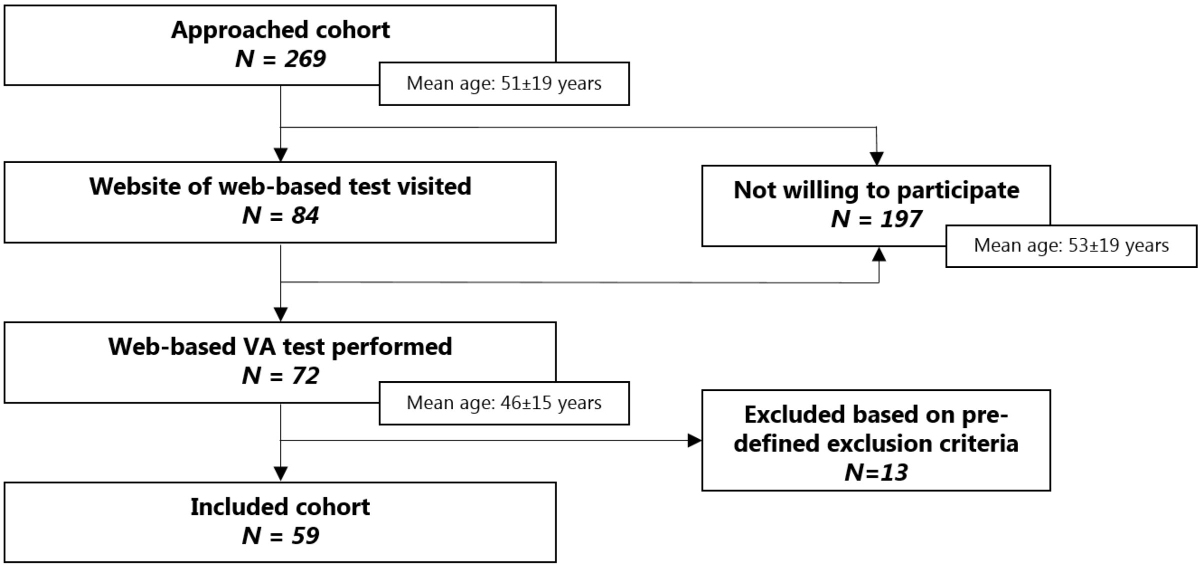

Supplementary Figure 1: Flowchart showing patient recruitment, including the mean (±SD) ages of the indicated groups. A total of 269 patients were invited to participate. Of the 84 patients who visited the website, 12 did not perform the test. The reported reason for this was the requirement to create an account on the company's website in order to collect and store the data. All 72 patients who started the test, completed it. No difficulties or technical errors occurred. Note that the 72 patients who performed the test successfully were significantly younger than the 197 patients who were not willing to participate (P=.005).
