# Supplementary material for: The Accuracy of a Web-Based Visual Acuity Self-assessment Tool Performed Independently by Eye Care Patients at Home: Method Comparison Study
Source: JMIR Form Res. 2023 Jan 25;7:e41045. doi: 10.2196/41045 (PMC9909522; doi:10.2196/41045)
Supplement: Multimedia Appendix 2 [file formative_v7i1e41045_app2.pdf]

Multimedia Appendix 2

Supplementary Table 1*: Multivariable GEE analysis to investigate the associations between clinical variables and the visual acuity outcomes in logMAR*

	Difference between the VA scores of the web-based and conventional test, in logMAR[a]			VA score of the web-based assessment, in logMAR			VA score of the conventional test, in logMAR		
	B	95% confidence interval	P-value[b]	B	95% confidence interval	P-value[b]	B	95% confidence interval	P-value[b]
Sex[c]	0.02	-0.01 to 0.06	0.65	-0.04	-0.14 to 0.07	0.49	-0.05	-0.16 to 0.06	0.38
Age	0.00	0.00 to 0.00	0.87	0.00	-0.00 to 0.01	0.13	0.00	-0.00 to 0.01	0.16
Use of mydriatics	-0.03	-0.10 to 0.04	0.41	0.15	-0.20 to 0.49	0.41	0.11	-0.17 to 0.40	0.44
Visual acuity influencing comorbidities[d]	0.01	-0.02 to 0.05	0.70	0.06	-0.07 to 0.18	0.39	0.04	-0.08 to 0.17	0.49
Symptoms at time of appointment[e]	0.02	-0.03 to 0.10	0.28	0.10	-0.02 to 0.21	0.11	0.06	-0.07 to 0.18	0.36
Interval[f]	0.00	-0.00 to 0.01	0.37	0.00	-0.02 to 0.02	0.81	0.00	-0.01 to 0.02	0.82

B: beta value; VA = Visual acuity

a: absolute difference between the two VA assessments in logMAR

b: analyzed using a Generalized Estimating Equations to correct for inclusion of two eyes of one patient

c: 'female' as reference

d: visual acuity influencing comorbidities such as: (secondary) cataract, keratitis, scleritis, corneal lesion or history of pars plana vitrectomy

e: symptoms associated with active uveitis at time of the hospital consultation: pain, floaters, photophobia, visual loss

f: interval in days between the web-based assessment and manifest assessment
